# CistromeMeta: a large language model powered tool for automated ChIP-seq metadata extraction

**DOI:** 10.1093/bioinformatics/btag380

**Published:** 2026-06-13

**Authors:** Nicholas Piccaro, Myles Brown, Clifford Meyer

**Affiliations:** Department of Data Science, Dana-Farber Cancer Institute, Boston, MA, United States; Center for Functional Cancer Epigenetics, Dana-Farber Cancer Institute, Boston, MA, United States; Department of Medical Oncology, Dana-Farber Cancer Institute, Brigham and Women’s Hospital, and Harvard Medical School, Boston, MA, United States; Department of Data Science, Dana-Farber Cancer Institute, Boston, MA, United States; Department of Biostatistics, Harvard T.H. Chan School of Public Health, Boston, MA, United States

## Abstract

**Summary:**

Public repositories such as NCBI’s Gene Expression Omnibus (GEO) contain large numbers of ChIP-seq experiments, but their reuse is limited by heterogeneous free-text metadata describing target proteins, histone marks, cell lines, tissues, and disease states. We introduce CistromeMeta, a Python-based command-line tool that leverages large language models (LLMs) in a few-shot setting to automatically extract and standardize ChIP-seq metadata from GEO XML records without custom model training. The tool validates extracted terms against authoritative biological databases, including NCBI Gene, Harmonizome 3.0, AnimalTFDB 4.0, Cellosaurus, Experimental Factor Ontology, and Uberon, producing standardized outputs with official gene symbols and ontology identifiers for scalable metadata curation.

**Availability and implementation:**

The Python source code is freely available at https://github.com/nickpiccaro/CistromeMetaX. An archived version of the software is available through Zenodo at DOI: 10.5281/zenodo.20244834. The tool requires Python 3.6+ and an OpenAI API key.

## 1 Introduction

Public databases such as GEO now host tens of thousands of chromatin immunoprecipitation sequencing (ChIP-seq) datasets with substantial integrative potential for meta-analyses in regulatory genomics (Kodama *et al.* 2012, Barrett *et al.* 2013). However, metadata heterogeneity creates a major bottleneck because key experimental descriptors, including the immunoprecipitated target protein, cell type, tissue, and disease context, are often recorded in free text or inconsistent formats (Wang *et al.* 2019, Lim *et al.* 2021, Caliskan *et al.* 2023). Different laboratories use different nomenclature or omit crucial details, making manual curation labor-intensive and preventing systematic aggregation of datasets.

Traditional bioinformatics approaches for metadata parsing, including fixed-field extraction and keyword matching, struggle with the variability in GEO records (Barrett *et al.* 2012, Bernstein *et al.* 2017, Zou *et al.* 2022). Large language models (LLMs) offer a promising solution because of their natural language understanding capabilities. Recent work introduced ChIP-GPT, which fine-tuned a LLaMA model to extract ChIP-seq metadata with approximately 90–94% accuracy (Cinquin 2024). However, fine-tuning requires substantial computational resources, curated training data, and ongoing maintenance.

CistromeMeta takes an alternative approach: it harnesses general-purpose LLMs in a few-shot setting, eliminating training requirements while achieving high accuracy through careful prompt engineering and database validation (Kong *et al.* 2024, OpenAI 2026). The tool automatically extracts the target protein, such as a transcription factor or histone mark, and ontology terms describing biological context, including cell line, cell type, tissue, and disease. A core innovation is the integration of LLM-driven semantic parsing with a verification layer that validates every output against curated databases, addressing LLM limitations such as hallucinations while ensuring standardized outputs.

## 2 Implementation

**Figure 1:**
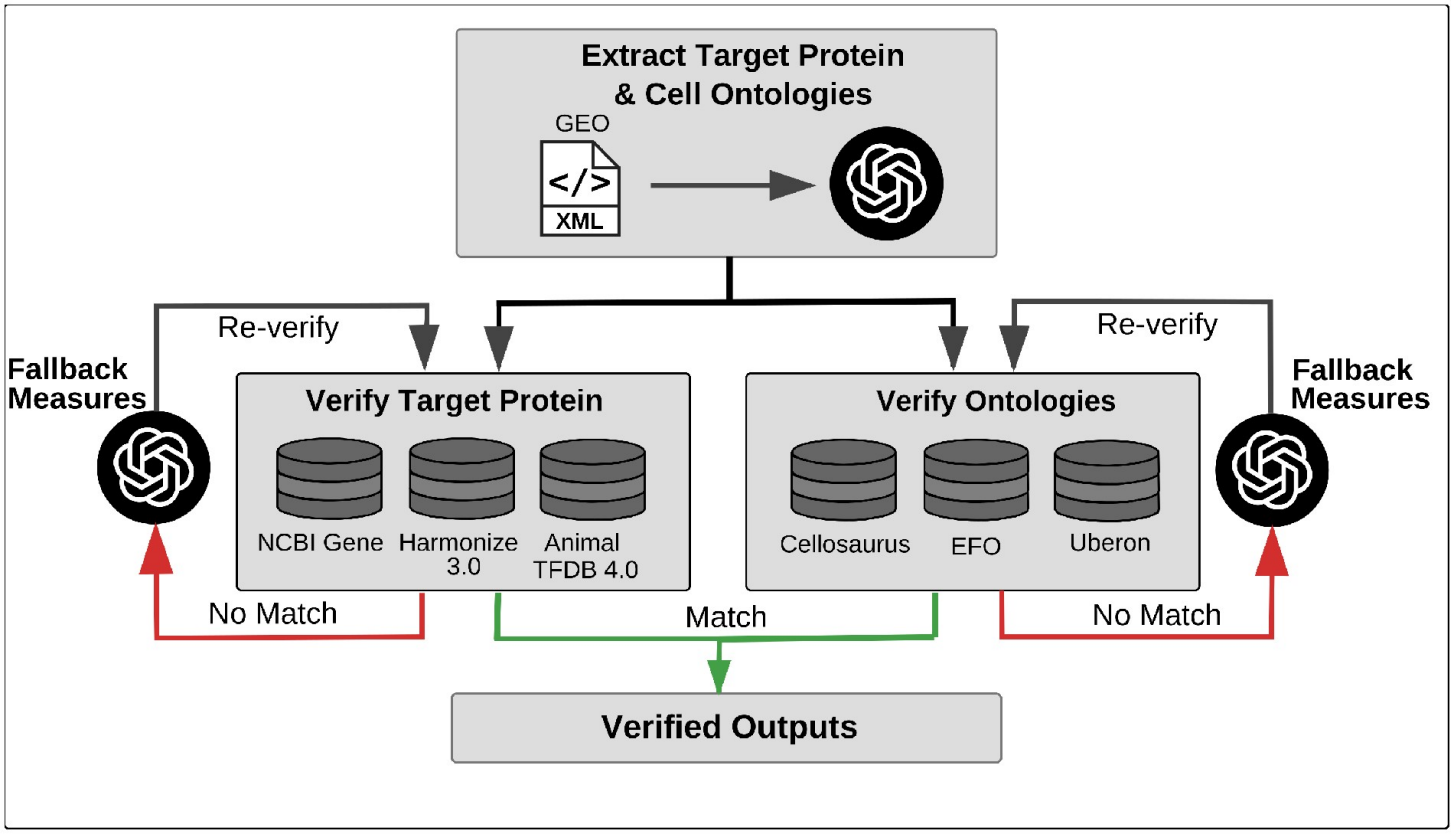
Simplified CistromeMeta architecture. The pipeline ingests GEO XML files, processes them through specialized LLM prompts with iterative fallback mechanisms, and validates results against biological databases to produce standardized JSON output.

CistromeMeta comprises an automated pipeline that ingests GEO metadata from GSM samples and GSE series, applies LLM-based parsing, and validates extracted information (Fig. 1). The tool uses a dual-pipeline architecture with separate extraction workflows for factors and ontologies, as separating these tasks proved more accurate than single-pass extraction.

We designed prompts to guide LLMs as specialized metadata extractors, framing the task as named entity recognition in genomics (Shanahan *et al.* 2023, Kong *et al.* 2024, Sivarajkumar *et al.* 2024, Wang *et al.* 2024, Chen *et al.* 2025). The system message provides specific constraints: output factors as official NCBI gene symbols, such as TP53, or standardized histone marks, such as H3K27ac; convert Roman numerals to numeric form; and preserve histone modification notation. We provide carefully selected few-shot examples targeting edge cases to ensure complete understanding, with structured JSON output for programmatic parsing. An iterative fallback mechanism addresses incomplete or ambiguous metadata by re-prompting with feedback from previous attempts and database validation failures.

For ChIP targets, we verify against NCBI Gene for official gene symbols, AnimalTFDB 4.0 for known transcription factors, and Harmonizome 3.0 for chromatin remodelers (Shen *et al.* 2023, O’Leary *et al.* 2024, Diamant *et al.* 2025). When a term returns multiple NCBI Gene matches, we prioritize AnimalTFDB’s human transcription factor subset, then Harmonizome’s chromatin remodeler subset, and finally employ a specialized LLM prompt to select the most likely candidate given sample context.

For biological source information, including cell line, cell type, tissue, and disease, we validate against Cellosaurus, EFO, and Uberon using fuzzy matching to account for phrasing variations (Malone *et al.* 2010, Mungall *et al.* 2012, Bairoch 2018). For example, K562 is matched to Cellosaurus entry CVCL_0004 and EFO : 0002067.

CistromeMeta is implemented in Python as both a command-line application and Python package. Ontology reference files in OBO or OWL format are pre-loaded into hashmap dictionaries containing essential name, identifier, and synonym pairs for rapid lookup. The validation pipeline implements a hierarchy in which transcription factors are considered more likely ChIP targets than chromatin remodelers and other classes. After hashmap-based database verification, the primary bottleneck is API request latency; full extraction and verification typically complete in under one second per sample.

The tool supports batch processing through JSON configuration files specifying GSM IDs, GSM-to-GSE mappings, and file paths. Users may alternatively provide a series of GSM or GSE accession IDs, in which case the tool automatically fetches the corresponding GEO XML records and runs the pipeline end-to-end without requiring pre-formatted inputs. Output is structured JSON containing validated factor information and mapped ontology terms with official accessions.

Each extracted factor is annotated with a classification, such as transcription factor, histone modification, chromatin remodeler, viral factor, bacterial gene-editing tool, gene, or none, and a status field flagging epitope-tagged constructs, control samples, incomplete epitope tags, or extraction and verification failures. Control samples, including input and IgG controls, are explicitly reported. Curated support is also included for common viral factors, such as LANA and EBNA3A, and bacterial proteins repurposed as genome-editing tools, such as Cas9.

## 3 Results

We evaluated CistromeMeta on 339 manually curated GEO ChIP-seq samples covering diverse target proteins, including CTCF, TP53, and NF-kB; histone modifications, including H3K27ac and H3K4me3; and biological contexts, many of which were not present in CistromeDB and represented more challenging or messier records. Without any prior fine-tuning, CistromeMeta achieved 98.2% accuracy (333/339) for factor identification and 97.6% (331/339) for cell line mapping (Table 1).

**Table 1:** CistromeMeta performance on benchmark datasets.

Metric	339 Samples	40 Samples (ChIP-GPT Subset)
Factor Accuracy	98.2% (333/339)	100% (40/40 vs. 92.5% ChIP-GPT)
Cell Line Accuracy	97.6% (331/339)	97.5% (39/40 vs. 95% ChIP-GPT)
Ontology Coverage*	100%*	100%*

* Percentage of samples where at least one ontology term was correctly identified when metadata was present.

On a 40-sample subset matching ChIP-GPT’s test set, after excluding 10 samples because of missing GEO IDs, CistromeMeta achieved 100% factor accuracy compared with 92.5% for ChIP-GPT, and 97.5% cell line accuracy compared with 95% for ChIP-GPT (Cinquin 2024). For comprehensive ontology extraction, including cell line, cell type, tissue, and disease, CistromeMeta successfully identified at least one matching ontology term in 100% of cases where such information was present in metadata (Table 1). On two additional focused samples independent of the 339-sample expanded benchmark, comprising 100 commonly studied ChIP-seq target proteins and 50 frequently used cell lines, CistromeMeta achieved perfect accuracy of 100/100 for factor identification and 50/50 for cell line mapping, respectively (Table S1 available as supplementary data at *Bioinformatics* online).

Errors primarily occurred when GEO records completely lacked target information or when available metadata context was ambiguous, internally inconsistent, or misleading. Token costs depend on model selection and the specific processing path used for each sample. Across tested models, factor extraction used approximately 5200 input tokens and 150 output tokens per sample on average, while ontology extraction used approximately 4730 input tokens and 400 output tokens per sample on average. Under current OpenAI GPT-5/GPT-5.1 pricing, this corresponds to roughly $2.94 per 10 000 samples for factor extraction, $5.41 per 10,000 samples for ontology extraction, and $8.35 per 10 000 samples when both steps are run (OpenAI 2026). These estimates reflect batch-processing conditions in which the system-prompt prefix is byte-identical across samples, allowing the pipeline to use provider-side prompt caching; for GPT-5/GPT-5.1, cached input pricing reduces from $1.25/M to $0.125/M input tokens. Costs still vary by sample type because control samples often terminate earlier, while samples requiring full extraction and database-aware verification use more tokens (Supplementary Tables S2 and S3, available as supplementary data at *Bioinformatics* online).

CistromeMeta’s implementation using LangChain allows straightforward model swapping (https://docs.langchain.com/oss/python/langchain/overview). This design makes switching between different models and providers seamless; users can plug in any LLM supported by LangChain. All reported benchmarks were performed using GPT-5.1; GPT-4o-mini remains the default model in the distributed package as a cost-efficient choice for large-scale batch processing (OpenAI 2026). As foundation models advance, CistromeMeta can inherit improvements without modification.

## 4 Conclusion

CistromeMeta demonstrates that general-purpose LLMs combined with domain-specific validation can achieve high accuracy compared with fine-tuned models while requiring no training infrastructure. The tool’s out-of-the-box approach leverages the broad pretraining of LLMs to understand varied phrasing and context; for example, it recognizes in UTA_ChipSeq_MCF-7_Pol2 that Pol2 is the target and MCF-7 is the cell line despite format variation.

Although CistromeMeta focuses on ChIP-seq, the methodology generalizes to other sequencing experiments. RNA-seq metadata, including tissue, treatment, and condition, or single-cell sequencing metadata could be extracted using similar LLM prompts paired with appropriate ontology validation. This suggests a broader paradigm in which bioinformatics pipelines incorporate LLM components for unstructured input handling alongside validation components that ensure outputs align with scientific databases.

The tool has limitations: it cannot recover information absent from GEO records, and iterative fallback mechanisms may occasionally validate semantically incorrect but database-matched terms. Dependency on external APIs could be addressed through on-premise LLM solutions or fine-tuning open models on our prompt instructions, although current GPT-4o-mini and GPT-5.1 capability proved sufficient without fine-tuning.

CistromeMeta complements existing efforts such as Cistrome Data Browser, MetaSRA, and TFmapper by providing rapid standardization for new entries and enabling users to create filtered datasets (Bernstein *et al.* 2017, Zeng and Li 2018, Taing *et al.* 2024). Future extensions will incorporate genetic modifications, drug treatments, antibody information, and confidence scoring with reasoning traces to aid debugging and enable human-in-the-loop validation.

In conclusion, CistromeMeta provides a practical, scalable solution to metadata heterogeneity in genomic repositories. By combining AI flexibility with database validation rigor, it achieves high accuracy while demonstrating low costs, delivering standardized JSON output with gene symbols and ontology IDs immediately usable for dataset integration. This work exemplifies how modern AI can accelerate scientific data curation, paving the way for AI-assisted bioinformatics infrastructure.

## Supplementary Material

btag380_Supplementary_Data

## Data Availability

The data underlying this article are available in the article and in its online supplementary material.
