# Supplementary material for: CistromeMeta: a large language model powered tool for automated ChIP-seq metadata extraction
Source: Bioinformatics. 2026 Jun 13;42(6):btag380. doi: 10.1093/bioinformatics/btag380 (PMC13294451; doi:10.1093/bioinformatics/btag380)
Supplement: btag380_Supplementary_Data [file btag380_supplementary_data.pdf]

Supplementary Table S1. CistromeMeta Performance on Focused Test Subsets

Test Subset	Task	N	Accuracy
Common ChIP-seq target proteins	Factor identification	100	100% (100/100)
Common cell lines	Cell line mapping	50	100% (50/50)

Performance on two focused subsets composed of widely studied ChIP-seq target proteins and commonly used cell lines. These samples are independent of the 339-sample expanded benchmark reported in Table 1.

Supplementary Table S2. Per-Sample Token Budgets in the CistromeMeta Pipeline

**Mode**	**Cached guidelines**	**XML payload**	**Wrapper**	**Total input**	**Output (non-thinking)**	**Output (thinking)**
Factor	4,500	~630	~70	~5,200	~150	~700
Ontology	4,000	~630	~100	~4,730	~400	~1,500
Both	8,500	~1,260	~170	~9,930	~550	~2,200

Per-sample input and output token budgets. The cached guidelines prefix is byte-identical across samples, enabling provider-side prompt caching after the first call. Output token counts are reported separately for non-reasoning and reasoning ("thinking") model modes.

Supplementary Table S3. Estimated Cost per 1,000 Samples (USD) Across Supported Providers

Provider	Model	$/M input (uncached → cached)	$/M output	Factor	Ontology	Both
OpenAI	gpt-5-nano	0.05 → 0.005	0.40	$0.12	$0.22	$0.34
OpenAI	gpt-4o-mini	0.15 → 0.075	0.60	$0.53	$0.65	$1.18
OpenAI	gpt-5-mini	0.25 → 0.025	2.00	$0.59	$1.08	$1.67
OpenAI	gpt-4.1-mini	0.40 → 0.10	1.60	$0.97	$1.33	$2.30
OpenAI	gpt-5 & gpt-5.1	1.25 → 0.125	10.00	$2.94	$5.41	$8.35
OpenAI	gpt-4.1	2.00 → 0.50	8.00	$4.85	$6.66	$11.51
OpenAI	gpt-4o	2.50 → 1.25	10.00	$8.88	$10.83	$19.70
Anthropic	Claude Haiku 4.5	1.00 → 0.10	5.00	$1.90	$3.13	$5.03
Anthropic	Claude Sonnet 4.6	3.00 → 0.30	15.00	$5.70	$9.39	$15.09
Anthropic	Claude Opus 4.5	5.00 → 0.50	25.00	$9.50	$15.65	$25.15
Google	Gemini 2.5 Flash-Lite	0.10 → 0.025	0.40	$0.24	$0.33	$0.57
Google	Gemini 2.5 Flash (thinking)	0.30 → 0.075	2.50	$2.30–3.30	$4.40–5.17	$6.70–8.47
Google	Gemini 3 Flash Preview (thinking)	0.50 → 0.125	3.00	$3.01–4.70	$5.37–6.87	$8.38–11.57

Estimated USD cost per 1,000 GSM samples by model and mode. List prices verified May 2026. Estimates assume warm-cache steady state, with first-sample costs amortized for batches of approximately 20 or more. GPT-5 rows use reasoning_effort="minimal". Gemini Flash ranges reflect cache-hit to cold-cache estimates and are approximate. Per-sample costs vary by sample type because controls often terminate early and histone-mark samples generally require less verification.
